# Effect of Swimming Exercise on Respiratory Muscle Strength and Respiratory Functions in Children with Autism

**DOI:** 10.5152/eurasianjmed.2023.22118

**Published:** 2023-06-01

**Authors:** Emine Adin, Zarife Pancar

**Affiliations:** 1 Department of Physical Education and Sports, Gaziantep University, Faculty of Sports Science, Gaziantep Turkey

**Keywords:** Exercise, strength, autism, respiratory, swimming

## Abstract

**Objective:**

The aim of this study is to examine the effects of swimming exercise on respiratory muscle strength and respiratory functions in children with autism. Autism is a mental disorder that affects many areas such as sensory, cognitive, motor, and psycho-motor development in individuals.

**Materials and Methods:**

For this purpose, 15 individuals with autism, 8 of which were in the experimental group and 7 in the control group, participated in the study. The experimental group was subjected to swimming exercise for 1 hour, 3 days a week, for 6 weeks. The control group was not included in this exercise. Respiratory muscle strength and pulmonary function tests were applied to both groups before and after the 6-week period. The obtained data were analyzed using Statistical Package for Social Sciences Program Version 22.0. Values were presented as minimum, maximum, mean, standard deviation, and standard error. The Shapiro-Wilk test was used to test for normality. Paired-sample *t*-test was used for pre-test and post-test, and independent-sample *t*-test was used for intergroup analysis.

**Results:**

At the end of 6 weeks, according to the statistical analysis data, there was a significant difference in some of the respiratory function parameters of the experimental group (*P* < .05), and an improvement was observed in the respiratory muscle strength values, but no significant difference was found (*P* > .05). No significant difference was found in the respiratory functions of the control group as a result of respiratory muscle strength measurements (*P* > .05).

**Conclusion:**

As a result, we can say that swimming exercise is effective in improving respiratory muscle strength and respiratory functions in children with autism.

**Main Points**

- As a result of the applied swimming exercise, improvements were observed in the respiratory functions of individuals with autism.
- The respiratory system is physiologically coordinated to meet the increased oxygen demand.
- The 6-week swimming exercise period is sufficient for the development of respiratory functions value in children with autism.

## Introduction

Autism is a mental disorder that affects many areas such as sensory, cognitive, motor, and psycho-motor development in individuals.^1^ They are unable or have difficulty in social interaction. It is very difficult for children with autism to communicate with other individuals other than their families. As a result of the researches, it is known that children with autism have looser muscle structure and their motor development progresses more slowly than healthy children.^2-^

It is known that physical activity has many psychological and health benefits for individuals. In its deficiency, different types of diseases such as cardiovascular diseases and various cancer types can be seen in individuals. These symptoms can also be seen in individuals with autism spectrum disorder (ASD), as well as creating great problems in areas such as socialization, communication, and meeting personal needs. In general, individuals with ASD lead a more sedentary and physically inactive life than individuals with normal development.^5-7^

Swimming is defined as a sport that aims to overcome or minimize the friction that prevents the movement in which the arms and feet work in coordination in the horizontal position on the water. Considering the pressure it creates on breathing, it can be said that the energy required for swimming is about 4 times the energy required for running, according to the energy data consumed by individuals who cover a distance by running and swimming.^8,9^ The respiratory system is very important in terms of daily life, work performance, and sportive performance capacity of the individual.^10-12^

In addition to the explanations above, it is thought that swimming exercise will have positive effects on the general health status of children with autism and, as the main determinant, on respiratory functions. The swimming exercise program should be prepared and implemented for these predetermined purposes. The aim of our study is to examine the effect of swimming exercise on respiratory muscle strength and respiratory functions in children with autism.

## Materials and Methods

The research was designed based on the control pretest–posttest design. Fifteen autistic children between the ages of 10 and 12 participated in the study. The subjects were divided into 2 groups as experimental (n = 8; 2 girls, 6 boys) and control (n = 7; 7 boys) groups. The respiratory functions and respiratory muscle strength measurements of the subjects were taken 1 day before and 1 day after the 6-week training program. Care was taken to form the subject groups, especially from individuals who did not do sports. The same pretest–posttest period was applied to the control group. This study was conducted in compliance with the ethical principles according to the Declaration of Helsinki, and it was approved by the local Institutional Review Board (Gaziantep University Ethics Committee, decision no: 221-2020).

After the first measurements, a 6-week swimming exercise program was applied to the experimental group. The control group was not included in this training program. The exercise program was set as 1 hour and 3 days a week with 1-day intervals for each individual. At the beginning of the training, warm-up and stretching movements were applied for 10 minutes, and the training was applied according to the ready exercise program in the remaining 50 minutes. At the end of each 20 minutes of training, 5 minutes of free play was given. The full participation of all individuals in the experimental group was ensured in the 6-week exercise program and the same program was applied to everyone.

### Pulmonary Function Tests

**Figure 1.**
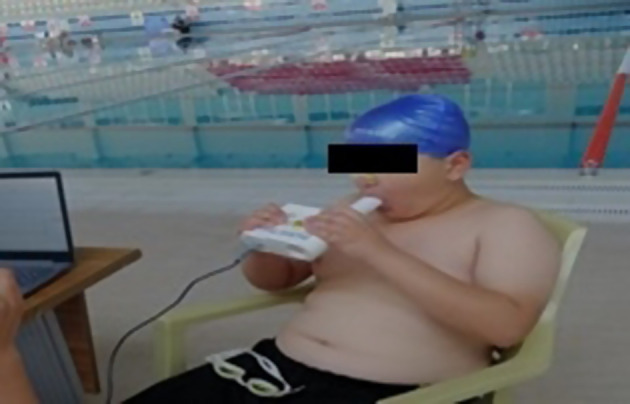
Forced vital capacity measurement.

*Forced vital capacity measurement* (Figure 1): Pocket Spiro USB-100 device was used. During the measurement, the subjects were allowed to wear light clothing. The subjects were told in advance that they had to do their best; otherwise, the results would be meaningless. A separate mouthpiece was carefully used for each subject. The subject's nose was blocked, and the mouthpiece was placed between the lips so that there was no gap in the corners of the mouth. During measurement, subjects first did 3 normal inspirations and exhalations, then inhaled at maximum force and rapidly, and then exhaled as quickly as possible to complete the measurement.^13-15^

**Figure 2.**
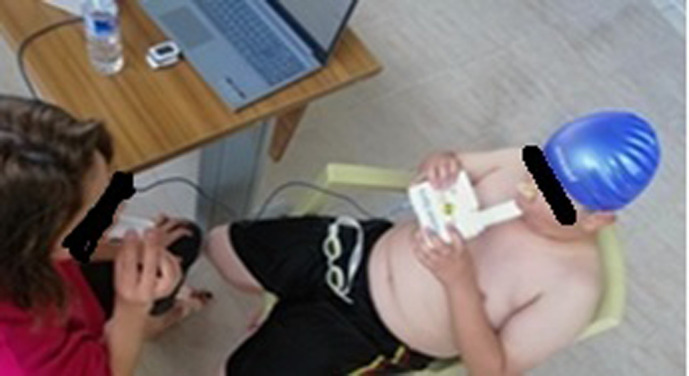
Vital capacity measurement.

*Vital capacity measurement* (Figure 2): During measurement, subjects first performed 3 normal inspirations and expirations, then completed the measurement by performing 1 slow maximum inspiration and 1 slow maximum expiration.^16^

*Breath inspiratory flow and peak expiratory flow calculation*: MEC Pocket Spiro MPM 100 electronic respiratory pressure gauge was used. The person is asked to make maximum expiration and maximum inspiration into the closed airway for 1-3 seconds. For peak expiratory flow (PEF), the person is given maximum inspiration and the person is asked to give maximum breath into the closed airway and continue this for 1-3 seconds. The measurement was repeated until the difference between the 2 best measurements was 10 cmH_2_O and the result of the difference in the measurements of the individuals was recorded as cmH_2_O.^17,18^

Statistical Package for Social Sciences Version 22.0 (IBM Corp., Armonk, NY, USA) program was used for statistical operations. The Shapiro–Wilk test was used to test for normality. The paired-sample *t*-test was used to compare pre- and post-test data, while the independent-sample *t*-test was used to compare the difference between pre-group and post-group tests.

## Results

In this study, which investigated the effects of swimming on respiratory muscle strength and respiratory functions in children with autism, a total of 15 autistic individuals, 8 of which were in the experimental group and 7 in the control group, were included.

The descriptive data of the subjects participating in the study are shown in Table 1. Experimental group forced vital capacity (FVC) mean was 1.86 ± 0.71 lt, control group FVC mean was 1.44 ± 0.27 lt, experimental group 1 second forced expiratory volume (FEV1) mean was 1.20 ± 1.04 lt, control group FEV1 mean was 1.15 ± 0.55 lt, experimental group FEV1/FVC mean was 66.25 ± 45.21%, control group FEV1/FVC mean was 83.43 ± 37.08%, mean PEF in the experimental group was 3.03 ± 1.48 lt/sec, mean PEF in the control group was 2.39 ± 0.84 lt/sec, mean PIF in the experimental group was 2.13 ± 0.86 lt/sec mean PIF in the control group was 1.89 ± 0.45 lt/sec, maximal voluntary ventilation (MVV) mean in the experimental group was 41.88 ± 36.40 lt/min, control group MVV mean was 40.27 ± 19.40 lt/min, experimental group vital capacity (VC) mean was 1.69 ± 0.48 lt, control group mean VC was 1.13 ± 0.55 lt, experimental group tidal volume (TV) mean was 0.56 ± 0.10 lt, control group TV mean was 0.52 ± 0.30 lt, the mean of the experimental group inspiratory vital capacity (IVC) was 1.09 ± 0.31 lt, and the mean of the control group IVC was 1.04 ± 0.56 lt.

Analysis of changes in respiratory function parameters as a result of swimming exercise is shown (Table 2).

While the pre-test FVC average of the experimental group was 1.86 ± 0.71 lt, the FVC average in the post-test was 2.50 ± 0.68 lt (Table 3). There was a significant difference between the pre- and post-test in the FVC value of the experimental group (*P* < .05). There was no significant difference between the pre-test and post-test in the FVC value of the control group (*P* > .05). There was a significant difference between the pretest and posttest in the FEV1 value of the experimental group (*P* < .05). There was no significant difference between the pre-test and post-tests in the FEV1 value of the control group (*P* > .05).

The analysis of the measurements taken from individuals with autism in the experimental-control groups in PEF and PIF values, which are respiratory muscle strength parameters, are given in Table 4.

## Discussion

This study was conducted to examine the effects of swimming on respiratory muscle strength and respiratory function in children with autism. At the end of the 6-week exercise program, respiratory function and respiratory muscle strength were measured to determine whether the applied training program was effective or not.

In physical activities, the oxygen demand of the muscles increases and the respiratory system is physiologically coordinated to meet the increased oxygen demand. Increasing respiratory function according to the intensity and duration of breathing exercises; the development of respiratory muscles depends on the expansion ability of the lungs and chest cavity, the elasticity of the bronchi and bronchioles.^19^ As a result of the applied swimming exercise, improvements were observed in the respiratory functions of individuals with autism. Considering the pre-test and post-test data obtained, a significant difference was found in the FVC, FEV1, MVV, and IVC values. An increase was observed in FEV1, FVC, VC, and TV values, but no significant difference could be obtained. No significant difference was detected in the respiratory parameters of the control group (*P* > .05).

Another study examined the effect of swimming on children’s respiratory parameters. A 6-week swimming training was given to the male swimmers (34 children aged 6-14 years) in the experimental group. It was concluded that FVC value was 6%, FEV1 value was 6.32%, MVV value was 6.52%, and FEF value was 20%-32% higher than that before the study.^20^ In a study, as a result of 8-week swimming training, the VC value of the experimental group was 4.90 ± 1.20 lt in the pre-test and increased to 6.60 ± 1.20 lt in the post-test.^21^ In different studies, a significant difference was found in the FVC, FEV1, FIV1, IC, and MVV values of the 8-12 age group as a result of 8 weeks of swimming exercise. Different from our age groups, 11 young women who study at the sports academy and do regular swimming training and 40 young women who do not train participated. In a study investigating the effect of 12-week swimming training on respiratory parameters, VC, FVC, and FEV1 values were found to be significantly different between women who did swimming training and women who did not.^22^

In a study conducted with adolescent swimmers, the pre-test and post-test values of VC, FVC, FEV1 parameters were found to be statistically significant among 310 elite swimmers aged 12-14 years for the spirometric evaluation of the respiratory system.^23^ Respiratory muscles are an important criterion in revealing performance. It was also supported by sports experts. The endurance of the respiratory muscles is important in building resistance to fatigue.^24-26^

In order to determine whether autistic individuals participating in the study had a positive effect on respiratory muscle strength, the difference between the 2 measurements was checked by measuring PIF and PEF values during the study. As a result of this measurement, no significant difference could be reached between the data obtained from the experimental group and the control group. In a different study, it was shown that an 8-week regular core training program strengthened the respiratory muscles and reduced the fatigue of the respiratory muscles due to the resistance caused by the bicycle.^27-29^

A total of 16 individuals with Down syndrome underwent 4-week inspiratory muscle training, and respiratory muscle strengths were measured before and after the training. In the data obtained, the mean PEF value of the experimental group before the test was 1.22 ± 0.32 lt/s, while the mean PEF value after the test increased to 1.73 ± 0.59 lt/s and 43.96 ± 45.30%. In the experimental group, the pre-measured PIF mean value increased 1.02 ± 0.27 lt/s, and the post-measurement PIF mean value increased to 1.32 ± 0.40 lt/s and 43.54 ± 79.85%. No significant difference was found in the PIF and PEF values of the control group.^11^

In a different study investigating the effect of swimming training on respiratory parameters, there was no significant difference in PEF values between women who did and did not train,^22^ and in another study, it was investigated whether the breathing exercises performed by children of the same age during the 8-week study period had an effect on vital capacity. As a result of the study, it was observed that the FVC, FEV1, PIF, and FEV1/FVC values of the swimmers did not change.^25^

Swimming exercises are effective for the performance of autistic children. However, since the exercises should be one-to-one work, effective and sufficient time is needed. Longer duration or regular swimming exercise may be recommended for autistic children. In our study, we worked with a small number of autistic children and limited its duration. By increasing the number of researchers and the number of children, respiratory functions can be improved by swimming for a long time. At the same time, the effects of exercise on social behavior changes can be tested.

A limited number of autistic children were included in our study. Larger number can be included. Conducting a separate study with each autistic child made the study difficult in terms of time and measurements and caused it to be prolonged. Participation of autistic children in swimming activities brought positive results in terms of both performance and social aspects.

As a result, we can say that swimming exercise performed in line with the goal of our study provides improvements that support the development of respiratory functions of individuals with autism. The endurance of the respiratory muscles is important in building resistance to fatigue. Therefore, swimming exercises and respiratory muscle activities should be supported in children with autism.

## Figures and Tables

**Table 1.** Descriptive Parameters of the Subjects

		Min.	Max.	Mean	SD
Experimental group (n =8)	Age (years)	10.00	11.00	10.25	0.46
	Height (cm)	130.00	153.0	142.8	7.53
	Body weight (kg)	31.00	53.00	40.63	9.72
	BMI (kg/m^2^)	15.40	23.60	19.68	3.11
	FVC (lt)	0.96	2.96	1.86	0.71
	FEV1 (lt)	0.00	2.78	1.20	1.04
	FEV1/FVC (%)	0.00	100.0	66.25	45.21
	PEF (lt/sn)	0.46	4.56	3.03	1.48
	PIF (lt/sn)	0.65	3.27	2.13	0.86
	MVV (lt/dk)	0.00	97.20	41.88	36.40
	VC (lt)	1.02	2.40	1.69	0.48
	TV (lt)	0.41	0.73	0.56	0.10
	IVC (lt)	0.76	1.53	1.09	0.31
Control group (n = 7)	Age (years)	9.00	12.00	10.57	1.27
	Height (cm)	138.00	160.0	145.2	7.97
	Body weight (kg)	34.00	50.00	40.86	6.18
	BMI (kg/m^2^)	14.80	25.00	19.46	3.32
	FVC (lt)	1.12	1.84	1.44	0.27
	FEV1 (lt)	0.00	1.71	1.15	0.55
	FEV1/FVC (%)	0.00	100.0	83.43	37.08
	PEF (lt/sn)	1.46	3.90	2.39	0.84
	PIF (lt/sn)	1.20	2.32	1.89	0.45
	MVV (lt/dk)	0.00	59.90	40.27	19.40
	VC (lt)	0.00	1.72	1.13	0.55
	TV (lt)	0.00	0.85	0.52	0.30
	IVC (lt)	0.00	1.72	1.04	0.56

FEV1, 1 second forced expiratory volume; FVC, forced vital capacity; IVC, inspiratory vital capacity; MVV, maximal voluntary ventilation; PEF, peak expiratory flow; PIF, breath inspiratory flow; SD, standard deviation; TV, tidal volume; VC, vital capacity.

**Table 2.** Analysis of Respiratory Function Values

Characteristics		Mean	SD	*P*
FVC (lt)	Pre-test	1.66	0.57	.156
	Post-test	1.94	0.85	
FEV1 (lt)	Pre-test	1.17	0.82	.072
	Post-test	1.72	1.03	
FEV1/FVC (%)	Pre-test	74.27	41.11	.362
	Post-test	85.27	34.72	
MVV (lt/dk)	Pre-test	41.13	28.72	.073
	Post-test	60.15	35.96	
VC (lt)	Pre-test	1.43	0.57	.167
	Post-test	1.62	0.57	
TV (lt)	Pre-test	0.54	0.21	.135
	Post-test	0.66	0.18	
IVC (lt)	Pre-test	1.06	0.43	.096
	Post-test	1.30	0.42	

FEV1, 1 second forced expiratory volume; FVC, forced vital capacity; IVC, inspiratory vital capacity; MVV, maximal voluntary ventilation; PEF, peak expiratory flow; PIF, breath inspiratory flow; SD, standard deviation; TV, tidal volume; VC, vital capacity.

***P*** **< .05.**

**Table 3.** Comparison of Pulmonary Function Values Between Pre–Post Test and Groups

			Mean	SD	*P*
Experimental group (n = 8)	FVC	Pre-test	1.86	0.71	.030
		Post-test	2.50	0.68	
	FEV1	Pre-test	1.20	1.04	**.017**
		Post-test	2.24	1.02	
	FEV1/FVC	Pre-test	66.25	45.21	.197
		Post-test	85.25	34.61	
	MVV	Pre-test	41.88	36.40	**.017**
		Post-test	78.26	35.53	
	VC	Pre-test	1.69	0.48	.209
		Post-test	1.93	0.63	
	TV	Pre-test	0.56	0.10	.127
		Post-test	0.72	0.23	
	IVC	Pre-test	1.09	0.31	**.025**
		Post-test	1.43	0.46	
Control group (n = 7)	FVC	Pre-test	1.44	0.27	.537
		Post-test	1.31	0.50	
	FEV1	Pre-test	1.15	0.55	.957
		Post-test	1.13	0.70	
	FEV1/FVC	Pre-test	83.43	37.08	.931
		Post-test	85.29	37.62	
	MVV	Pre-test	40.27	19.40	.953
		Post-test	39.46	24.64	
	VC	Pre-test	1.13	0.55	.528
		Post-test	1.28	0.23	
	TV	Pre-test	0.52	0.30	.574
		Post-test	0.60	0.09	
	IVC	Pre-test	1.04	0.56	.668
		Post-test	1.16	0.33	

FEV1, 1 second forced expiratory volume; FVC, forced vital capacity; IVC, inspiratory vital capacity; MVV, maximal voluntary ventilation; PEF, peak expiratory flow; PIF, breath inspiratory flow; SD, standard deviation; TV, tidal volume; VC, vital capacity.

***P*** **< .05.**

**Table 4.** Comparison of Respiratory Muscle Strength (PEF, PIF) Pre–Post Test Difference

			Mean	SD	*t*	*P*
Experimental group (n = 8)	PEF	Pre-test	3.03	1.48	−1.549	.165
		Post-test	3.75	0.76		
	PIF	Pre-test	2.13	0.86	0.147	.887
		Post-test	2.09	0.38		
Control group (n = 7)	PEF	Pre-test	2.39	0.84	−0.139	.894
		Post-test	2.42	0.7		
	PIF	Pre-test	1.89	0.45	0.088	.933
		Post-test	1.87	0.44		

PEF, peak expiratory flow; PIF, breath inspiratory flow.

***P*** **< .05.**
